# Multifaceted Biological Activity of Selected Flavone *C*-Monoglucosides

**DOI:** 10.3390/ijms262010124

**Published:** 2025-10-17

**Authors:** Danuta Zielińska, Henryk Zieliński

**Affiliations:** 1 Department of Chemistry, University of Warmia and Mazury in Olsztyn, Plac Łódzki 4, 10-721 Olsztyn, Poland; danuta.zielinska@uwm.edu.pl; 2 Team of Chemistry and Biodynamics of Food, Institute of Animal Reproduction and Food Research, Polish Academy of Sciences, Trylińskiego 18, 10-683 Olsztyn, Poland

**Keywords:** flavone *C*-monoglucosides, quercetin, angiotensin converting enzyme inhibition, acetylcholinesterase enzyme inhibition, antiglycation activity, antioxidant activity, differential pulse voltammetry

## Abstract

Determination of the multifaceted activity of selected flavone *C*-monoglucosides, namely orientin (OR), homoorientin (hOR), vitexin (VT), and isovitexin (iVT) in comparison to quercetin (Q) was addressed. Their antioxidant activity was characterized by the peak oxidation potentials ($E_{pa}$) provided by the differential pulse voltammetry (DPV) method, whereas their inhibitory activity towards angiotensin converting enzyme (ACE), acetylcholinesterase (AChE), and advanced glycation end-products (AGEs) formation was measured in a model system. The relationship between the multifaceted activity of flavone *C*-monoglucosides and their $E_{pa}$ was evaluated. The rank of the antioxidant activity in comparison to quercetin was Q > hOR ≈ OR > iVT ≈ VT, whereas the order of the ACE inhibitory activity was Q > hOR > OR > VT > iVT. The correlation between IC_50_ for ACE inhibition and E_pa_ values was r = 0.787. This finding was confirmed by the negative correlation between ACE inhibitory activity and antioxidant activity of these compounds (r = −0.838). The order of the AChE enzyme inhibitory activity was hOR > OR > iVT > VT > Q, whereas the rank of anti-AGEs activity was hOR > OR > iVT > Q > VT > AG (aminoquanidine), and the weak positive correlation between IC_50_ and E_pa_ was noted (r = 0.546 for BSA (bovine serum albumin)/glucose system and r = 0.580 for BSA/methylglyoxal system). In contrast, the anti-AGEs activity was negatively correlated with IC_50_ values for ACE inhibition (r = −0.669), whereas no correlation was found between the ACE and AChE inhibitory activities. The results expand our knowledge of the multifaceted activity of flavone *C*-monoglucosides.

## 1. Introduction

Food of plant origin is a source of phenolic compounds with beneficial actions towards the prevention or delay of degenerative diseases [1,2]. Flavonoids, which are natural phenolic compounds derived from the secondary plant metabolism, are the most vital phytochemicals in diets [3].

Flavones from plants are typically conjugated as 3-O and 7-O-glycosides and may also have acetyl or malonyl moieties [4]. Flavone C-glycosides are most commonly detected as 6-C- and 8-C-glucosides [5,6]. There is limited information about the health benefits of flavonoid *C*-glycosides [7,8], especially about orientin (OR), homoorientin (hOR), vitexin (VT), and isovitexin (iVT) [7,9]. In this context, the multifaceted activity of OR, hOR, VT, and iVT in comparison to quercetin (Q) has attracted attention due to their natural high content in fruits and vegetables [5,6,9].

Orientin (OR) and homoorientin (hOR) and their 4′-deoxy analogs, vitexin (VT) and isovitexin (iVT), are the main flavone *C*-monoglucosides. They were found in the *Pterocarpus marsupium* tree, the fruits of *Cucurbitaceae,* in common (*Fagopyrum esculentum* Moench) and Tartary buckwheat (*Fagopyrum tataricum* Gaertn.) seeds and sprouts, and in *Atractylodes japonica* leaves [10,11,12,13,14]. Moreover, VT and iVT are the main flavone *C*-monoglucosides in mung bean [15], bamboo, and pigeon pea leaves [16,17]. The health-beneficial actions of flavone C-monoglucosides were recently described [6,7,18]; however, their inhibitory activity against acetylcholinesterase (AChE), angiotensin-converting enzyme (ACE), and advanced glycation end-products (AGEs) formation was not reported.

The electrochemical methods, such as cyclic voltammetry (CV), differential pulse voltammetry (DPV), and square wave voltammetry (SWV) for the determination of the antioxidant activity have emerged in the past two decades [18,19,20]. Differential pulse voltammetry (DPV) is a technique useful for the characterization of electroactive molecules under the specified experimental conditions [21]. The voltammetric data provide qualitative information (peak of anodic oxidation potential (Epa) indicating antioxidant strength) and quantitative information (peak currents or charges calculated in predefined potential ranges) proportional to antioxidative activity. The DPV is an excellent technique to determine the Epa. The low value of Epa corresponds to the high antioxidant power [22,23].

Hypertension seriously affects the world’s adult population, and it is a risk factor for the development of cardiovascular disease (CVD) [24,25,26]. Hypertension is often treated with synthetic drugs that inhibit angiotensin-I converting enzyme [27]; however, their action is often accompanied by unbeneficial side effects such as skin rashes, dry cough, hypotension, and angioedema [28]. For this reason, searching for alternative natural inhibitors is of great interest, and screening the ACE inhibitory activities of flavone C-monoglucosides may provide new knowledge for the verification of the functional properties.

The acetylcholine esterase inhibitors, which are able to increase the amount of the neurotransmitter acetylcholine (ACh) in the brains of the elderly population, are the most promising pharmacotherapy for the treatment of Alzheimer’s disease (AD) and related neurological disorders [29,30,31,32]. The most well-known biologically active inhibitors of plant origin include alkaloids, flavonoids, and phenolic compounds; however, no information is available on the inhibitory activity of flavone C-monoglucosides [33,34,35,36].

The formation of advanced glycation end-products (AGEs) is a risk factor associated with ageing for diabetes, uremia, cataract, atherosclerosis, and Alzheimer’s disease [37,38]. Both the dietary AGEs derived from thermally processed food and those generated endogenously in the human organism are the most important proinflammatory compounds [39]. Their inhibitory activity against AGEs formation is generally based on the scavenging of the free radicals generated in the glycation reaction [15,40,41,42]. 

**Figure 1:**
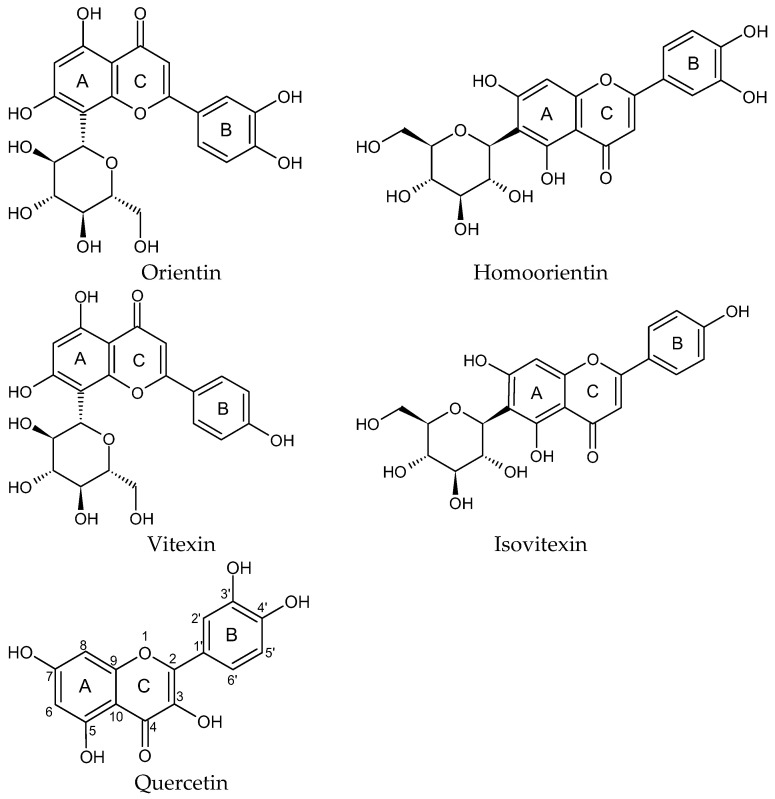
Structures of the investigated compounds: Orientin (OR), Homoorientin (hOR), Vitexin (VT), Isovitexin (iVT), and Quercetin (Q).

The aim of the study was to demonstrate the multifaceted activity of orientin (OR), homoorientin (hOR), vitexin (VT), and isovitexin (iVT) in comparison to quercetin (Q) (Figure 1). The relationship between the antioxidant activity of these compounds and their inhibitory activity against ACE, AChE, and AGEs formation was addressed.

## 2. Results

### 2.1. The Anodic Oxidation Potentials of Selected Flavone C-Monoglucosides in Comparison to Quercetin Provided by the Differential Pulse Voltammetry

**Figure 2:**
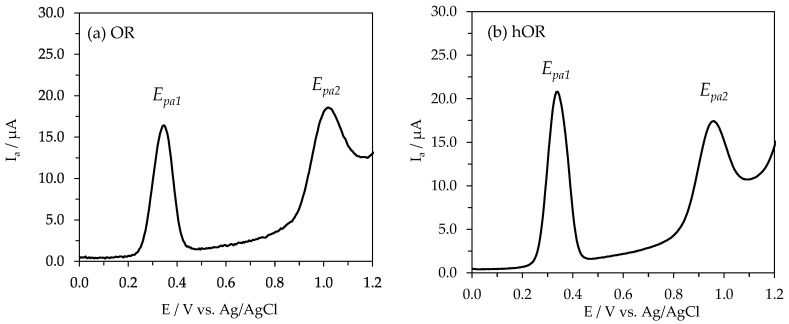

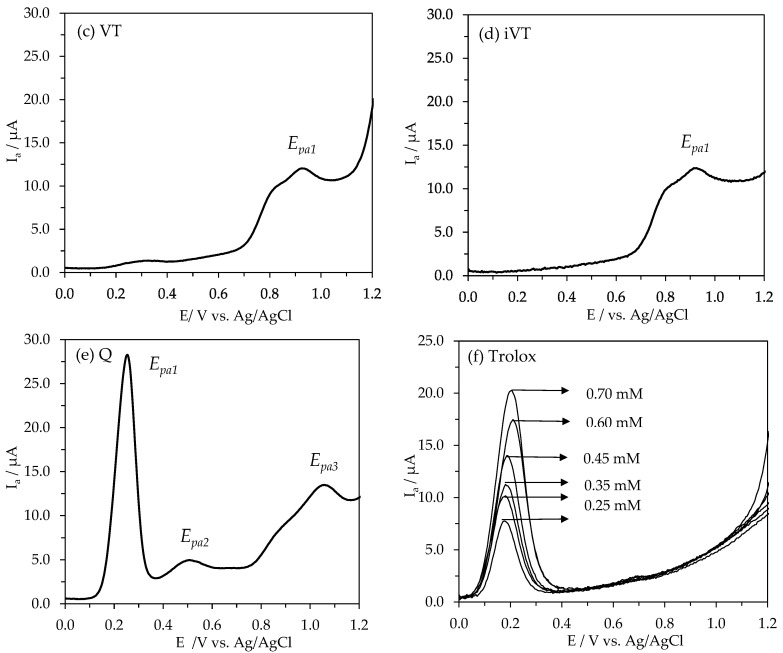
Differential pulse voltammograms of 0.25 mM standard solutions of: (**a**) OR, (**b**) hOR, (**c**) VT, (**d**) iVT, (**e**) Q, and (**f**) Trolox solutions in the concentration range from 0.15 to 0.70 mM.

Previously, the electrochemical behavior of quercetin has been studied; however, scarce information is related to the flavone *C*-monoglucosides [18,43,44]. In this study, the electroactivity of the selected flavone *C*-monoglucosides in comparison to quercetin is characterized by the first oxidation potentials (E_pa1_). The area under the anodic peaks was calculated to express their antioxidant activity, as was previously suggested [18,45,46]. The recorded differential pulse voltammograms are shown in Figure 2.

The OR, hOR, VT, iVT, and Q showed well-defined oxidation peaks with the first peak potentials of 345, 336, 925, 926, and 255 mV (vs. Ag/AgCl) for 0.25 mM standard solutions in 0.1 mM acetate-acetic buffer (pH 5.0) in 80% methanol (Table 1).

The order of the antioxidant activity of flavone *C*-monoglucosides in comparison to quercetin was Q > hOR ≈ OR > iVT ≈ VT. The antioxidant activity of OR and hOR was about threefold higher than compared to VT and iVT; however, it was only lower by 21% and 15% in relation to the antioxidant activity of Q. Having this rank, the question regarding the impact of the antioxidant activity of flavone *C*-monoglucosides on their multi-faceted biological activities was further addressed. Captopril and GSH voltammograms were not provided in our study since their chemical structures and lack of hydroxyl groups did not allow us to compare the results to those provided for flavone *C*-monoglucosides. Captopril’s chemical structure includes an L-proline derivative with a sulfhydryl group, making it a potent ACE inhibitor. This thiol group is crucial for its electroactivity, allowing it to be detected electrochemically and undergo reactions like oxidation. However, the gold electrode in the DPV technique, instead of the glassy carbon electrode, is recommended, and the electroactivity of captopril was reported in detail [47]. Gutathione (GSH) also exhibits significant electrochemical activity, particularly in its reduced form, and can be detected electrochemically through various methods like differential pulse voltammetry (DPV). It can also be oxidized electrochemically on modified electrodes, allowing for the detection and quantification of GSH. The standard redox potential of GSH is around −0.23 V vs. NHE.

### 2.2. Angiotensin-I-Converting Enzyme Inhibitory Activity of Selected Flavone C-Monoglucosides in Comparison to Quercetin

The ACE inhibitory activity of selected flavone *C*-monoglucosides in comparison to quercetin is shown in Table 2. The IC_50_ values for ACE inhibition of flavone *C*-monoglucosides in comparison to quercetin ranged from 59.29 µM for Q to 161.31 µM for iVT as compared to glutathione (IC_50_ = 41.68 µM) and captopril (IC_50_ = 0.0059 µM). Lower IC_50_ values were noted in conjunction with higher ACE inhibitory activity. The IC_50_ values of flavone *C*-monoglucosides were significantly different (*p* < 0.05) based on the one-way analysis of variance (ANOVA) (Table 2). The ACE inhibitory activity of OR was lower by 6.9% as compared to hOR, whereas the ACE inhibitory activity of VT was higher by 19.6% in comparison to iVT. The order of the ACE inhibitory activity of flavone C-monoglucosides in comparison to quercetin was Q > hOR > OR > VT > iVT. The IC_50_ of flavone *C*-monoglucosides was twice to threefold higher than that value for quercetin and for the small tripeptide reduced glutathione (GSH), thus simply indicating that the ACE inhibitory activity of flavone C-monoglucosides was twice to threefold lower.

The IC_50_ value represents the concentration of each compound that inhibits ACE activity by 50%. A lower IC_50_ value indicates higher ACE inhibitory activity. The correlation coefficient between ACE inhibitory activities, expressed as IC_50_, and E_pa_ values was r = 0.787, indicating a clear relationship between the chemical structure and antioxidant activity and ACE inhibitory activity. This finding was confirmed by the negative correlation between ACE inhibitory activity and antioxidant activity of these compounds (r = −0.838). The lower IC_50_ values were associated with the higher antioxidant activity of the flavone *C*-glucosides. However, in comparison to the pharmaceutical ACE inhibitor captopril, flavone *C*-monoglucosides and quercetin showed low ACE inhibitory activity.

### 2.3. Acetylcholinesterase Inhibitory Activity of Selected Flavone C-Monoglucosides in Comparison to Quercetin

The IC_50_ values of selected flavone *C*-glucosides for AChE inhibition are presented in Table 3.

The IC_50_ values for AChE inhibition ranged from 0.581 µM to 1.374 µM in comparison to galanthamine (IC_50_ = 0.043 µM). A lower IC_50_ value indicates higher AChE inhibitory activity. Homoorientin and orientin showed the highest AChE inhibitory activity, which was about 14–20 times lower than that of galanthamine. The AChE enzyme inhibitory activity of hOR was the highest among flavone C-monoglucosides, as indicated by the lowest IC_50_ value. The order of the AChE enzyme inhibitory activity was galanthamine > hOR > OR > iVT > VT > Q, thus indicating a weak relationship with the chemical structure. Galanthamine was taken as a reference compound since it is licensed in Europe for AD treatment [48].

### 2.4. The Inhibitory Activity of Selected Flavone C-Monoglucosides in Comparison to Quercetin Against the Advanced Glycation End Products (AGEs) Formation

The inhibitory activity of selected flavone *C*-monoglucosides in comparison to quercetin against AGEs formation is shown in Table 4.

The IC_50_ values against AGEs formation in the BSA/glucose model system ranged from 0.014 to 0.255 mM as compared to aminoquanidine (IC_50_ = 0.432 mM). The antidiabetic drug, namely aminoguanidine, which is a hydrazine derivative, showed systemic toxicity upon long-term administration, presumably due to its potent inhibition of catalase (and inducible nitric oxide synthase. The rank of anti-AGEs activity in the BSA/glucose model system was hOR > OR > iVT > Q > VT > AG. The anti-AGEs activity of hOR was about fivefold higher in comparison to OR, about 18, 15, and 17 times higher in relation to VT, iVT, and Q, and finally about 1.313 × 10^3^ times higher in relation to AG. Similarly, the IC_50_ values against AGEs formation in the BSA/MGO model system were noted from 0.3255 to 0.466 mM in relation to the IC_50_ of AG (0.531 mM). The rank of anti-AGEs activity in the BSA/MGO model system was OR > hOR > iVT > VT > Q > AG. The anti-AGEs activity of OR, hOR, iVT, VT, and Q was higher by 39, 26, 23, 18, and 12% in relation to AG.

## 3. Discussion

Currently, the UV–Visible spectrophotometry is usually employed for the determination of the antioxidant activity of compounds or the antioxidant capacity of food samples [48,49]. In the ABTS, FRAP, and Folin–Ciocalteu Reagent methods, the mechanism of action is the transfer of a single electron or capture of a synthetic radical according to the used method (SET); while the mechanism of action of the ORAC method is the transfer of hydrogen atoms (HAT). However, these assays have been criticized as the ABTS•+ radical and DPPH• are not representative of biomolecules and are not even found in any biological system. Moreover, the Folin–Ciocalteu Reagent method is not applicable for lipophilic compounds/matrices. The limitation of the FRAP assay is connected with the redox potential of any compound, which is lower than that of the redox pair Fe(III)/Fe(II). Such compounds may theoretically reduce Fe(III) to Fe(II), contributing to the FRAP value and inducing falsely high results [19]. In the photochemiluminescence assay (PCL), the photochemical generation of free radicals is combined with the sensitive detection by using chemiluminescence. The PCL is based on the photo-induced autoxidation inhibition of luminol by antioxidants, mediated by the radical anion superoxide, and is suitable to measure the radical scavenging properties of single antioxidants as well as more complex food systems in the nanomolar range [50]. The differential pulse voltammetry (DPV) provides valuable information on the electroactivity of redox-active substances [23,51] due to their direct electron-donating capacity, and the obtained oxidation peak potentials (Epa) are related to the antioxidant strength [52,53]. The redox-active substances typically include flavonoids, phenolic acids, some vitamins, and Maillard reaction products with reducing power. Recently, DPV was also used to evaluate the antioxidant capacity in different food matrices [54,55]. The applied DPV technique is not affected by the turbidity or color of the sample and, therefore, can be used as an environmentally friendly tool for the determination of the antioxidant capacity in food. 

The provided rank of the antioxidant power of flavone *C*-monoglucosides in comparison to quercetin was Q > hOR ≈ OR > iVT ≈ VT. This order reflects the current knowledge on the structural features and the nature of substitutions on rings B and C, determining the antioxidant activity of flavonoids [56,57]. The structural features include the degree of hydroxylation as well as the positions and number of the –OH groups in the B ring, as well as the double bond between C-2 and C-3, conjugated with the 4-oxo group in ring C, which enhances the radical scavenging capacity of flavonoids [57]. Moreover, substitution of the 3-OH in ring C results in an increase in torsion angle and loss of coplanarity, [58] whilst the presence of the hydroxyl groups in ring A and catechol structure or 4’-hydroxyl group in ring B seems to enhance the antioxidant activity [59].

Recently, Blasco et al. [60] proposed using the first oxidation potential of phenolic compounds to differentiate their antioxidant power. Taking this proposal, Q, OR, and hOR were classified as compounds with high antioxidant power (Ep < 0.4 V), while VT and iVT were classified as compounds with low antioxidant power (0.8 V < Ep < 1.3 V). 

Recently, a lot of bioactive compounds with potent ACE inhibitory activity have been separated and characterized from enzymatic hydrolysates of many foodstuffs, such as casein [61], tuna bone protein [62], soybean [63], lentil [64], apricot almond meal hydrolysate [65], and many other foods [66,67]. The phenolic compounds originated from plants showed ACE inhibitory activity [68,69,70], and additionally, they protected against oxidative stress [71] and inflammation [72]. In our study, the order of the ACE inhibitory activity Q > hOR > OR > VT > iVT indicated the importance of the structure–activity relationship. Our findings were in accordance with the study performed by Guerrero et al. [73] and Zielińska et al. [74], who provided data on the inhibitory ACE activity of selected flavonoids and their relationship to the structural basis. The high ACE inhibitory activity provided by the same analytical method as in our study was found for Q, as it was confirmed by Tsai et al. [75], whereas in this study, the ACE inhibitory activity of flavone C-monoglucosides is shown for the first time. These IC_50_ values of VT and iVT were only slightly higher compared to the ACE inhibitory activity of OR and hOR, thus indicating their lower ACE inhibitory activity due to the lack of the catechol group in the B-ring. These findings were in accordance with other observations that the flavonoid basic structure significantly affects their multifunctional activities [76]. This relationship was previously described for catechins and hydroxybenzoic acids [77,78]. The observed inhibitory effect on ACE activity in this study may be related to the number and position of hydroxyl groups. The impact of the interaction of phenolic compounds with the zinc ion, which is stabilized by other interactions with amino acids in the active site, was also reported as a factor influencing the ACE inhibitory activity [76]. 

During the last decade, alkylpyridinium polymers, dehydroevodiamine (DHED), alkaloids, and carbamate-type AChE inhibitors have been reported; however, due to bioavailability issues and potential side effects, there remains considerable interest in developing better AChE inhibitors [34,79]. This study presents the acetylcholinesterase inhibitory activity of flavone *C*-monoglucosides for the first time. The order of the AChE enzyme inhibitory activity was galanthamine > hOR > OR > iVT > VT > Q, thus indicating a weak relationship with the chemical structure. The principal action of galanthamine, a drug isolated from plants of the *Amaryllidaceae* family and recommended in Europe for AD treatment, is to provide symptomatic relief [80,81]. As Q, OR, and hOR were ranged as compounds with a high antioxidant potential, whilst VT and iVT were compounds with a low antioxidant power [60], their correlation with acetylcholinesterase inhibitory activity was investigated. In this study, no correlation between AChE inhibitory activities and values of E_pa_ and antioxidant activity was found (r = 0.319 and r = −0.245, respectively), thus clearly indicating the weak impact of the antioxidant potential of these compounds on AChE inhibitory activity.

Recently, flavonoids, aspirin, vitamin B1 and B6, and penicillamine have been reported as AGEs inhibitors partially responsible for the prevention of aging and diabetes complications [15,82]. Since reaction of proteins with reducing sugars as well as with short chain dicarbonyl metabolites, such as glyoxal and methylglyoxal, is responsible for the AGEs formation, the inhibitory effect of standard solution of orientin, homoorientin, vitexin, isovitexin and quercetin in comparison to aminoquanidine (AG) was measured in BSA/glucose (bovine serum albumin/glucose) and BSA/MGO (bovine serum albumin/methylglioxal) model systems. 

This study showed anti-AGEs activity of flavone *C*-glucosides, and the rank of anti-AGEs activity in the BSA/glucose model system was hOR > OR > iVT > Q > VT > AG, whereas in the BSA/MGO model system, it was OR > hOR > iVT > VT > Q > AG. In our study, a weak positive correlation was found between IC_50_ of OR, hOR, iVT, VT, and Q obtained in the BSA/glucose and BSA/MGO model system and their E_pa_ values provided by DPV. The correlation coefficient had a value r = 0.546 (BSA/glucose system) and r = 0.580 (BSA/MGO system). These results clearly indicate that OR and hOR display strong inhibitory capacity on the AGEs formation as compared to quercetin and aminoquinidine. Moreover, the IC_50_ of OR, hOR, iVT, VT, and Q obtained in the BSA/MGO model system was negatively correlated with their IC_50_ values for ACE inhibition (r = −0.669) and positively for AChE inhibition (r = 0.319), whereas no correlation was found between their ACE and AChE inhibition activities.

In future work, the development of nutritional products and semisynthetic analogs is envisaged.

## 4. Materials and Methods

### 4.1. Chemicals

Quercetin (Q; 3,3′,4′,5,7-pentahydroxyflavone), orientin (OR; 3′,4′,5,7-tetrahydroxyflavone-8-glucoside), homoorientin (hOR; 3′,4′,5,7-tetrahydroxyflavone-6-glucoside), vitexin (VT; 4′,5,7-trihydroxyflavone-8-glucoside) and isovitexin (iVT; 4′,5,7-trihydroxyflavone-6-glucoside) standards (HPLC-grade) were obtained from Extrasynthese Company Inc. (Lyon, France). Captopril, angiotensin-converting enzymes (ACE) from porcine kidneys (EC 3.4.15.1), 6-hydroxy-2,5,7,8-tetramethylchroman-2-carboxylic acid (Trolox), sodium azide, bovine serum albumin (BSA), D-glucose, methylglyoxal (MGO), and aminoguanidine hydrochloride were purchased from Sigma (Sigma Chemical Company, Saint Louis, MO, USA). A three-tris-base buffer was prepared as follows: buffer A: 300 mM Tris-base buffer (pH 8.3) with 2 mM ZnCl_2_; buffer B: 150 mM Tris-base buffer (pH 8.3); and buffer C: 150 mM Tris-base buffer (pH 8.3) with 1125 M NaCl. The substrate *o*-aminobenzoylglycyl-*p*-nitrophenylalanylproline (Abz-Gly-Phe(NO_2_)-Pro) was obtained from Bachem (Bubendorf, Switzerland). Acetylthiocholine iodide (ATCI), acetylcholinesterase (AChE), 5,5′[2-nitrobenzoic acid] (DTNB), and galantamine hydrobromide were obtained from Sigma Chemical Co. (Poznań, Poland). Methanol, acetic acid (supra-gradient), and sodium acetate were from Merck KGaA, Darmstadt, Germany. All other reagents of reagent-grade quality were from POCh, Gliwice, Poland. Water was purified with a Mili-Q system (Milipore, Bedford, MA, USA).

### 4.2. Measurement of the Anodic Oxidation Potentials of Flavone C-Monoglucosides in Comparison to Quercetin with the Differential Pulse Voltammetry

Electrochemical measurements were performed at room temperature (25 ± 2 °C) in a small-volume electrochemical cell using a potentiostat type SP-240 (BioLogic Science Instruments, Seyssinet-Pariset, France) controlled by EC-Lab V11.36 software. Differential pulse voltammograms were obtained with a pulse amplitude of 100 mV, a potential step of 5 mV, and a modulation time of 0.2 s. Four scans were registered for each sample, and the reported data correspond to the average of at least three replicates. The working electrode was a glassy carbon electrode, GCE (3 mm diameter, BASi M-2012, and the auxiliary and reference electrodes were platinum wire (BASi MW-1033) and Ag/AgCl (3 M KCl, BASi-MW-2052). All potentials are quoted against the used reference electrode. Before each scan, the surface of the glassy carbon electrode was polished on a polishing cloth with 0.05 μm alumina paste and ultrasonically rinsed in deionized water. After polishing, the electrode was washed with ultrapure water and dried with absorbent paper. DPV measurements were carried out in a standard solution of flavone *C*-monoglucosides and quercetin at a final concentration of 250 μM. Before measurements, the standard solutions were diluted in 0.2 M acetate-acetic buffer (in 80% methanol) at pH 5.0 (*v*/*v*: 1:1). Buffer solution also served as a supporting electrolyte [18,83]. For the test purpose, from registered DPV voltammograms, the anodic oxidation potentials (E_pa_) in mV and the integrated area under anodic peaks in μA were determined.

### 4.3. Determination of the Angiotensin-I-Converting Enzyme Inhibitory Activity of Flavone C-Monoglucosides

Angiotensin converting enzyme (ACE) inhibitory activity was performed according to the method of Sentandreu and Toldra [84]. The details of the procedure were described recently by Zielińska et al. [74]. Standards of OR (luteolin-8-C-glucoside), hOR (luteolin-6-C-glucoside), VT (apigenin-8-C-glucoside), iVT (apigenin-6-C-glucoside), and Q (quercetin) were dissolved in 80% methanol. Each standard was diluted to various concentrations using deionized water. The multiscan microplate fluorometer (Tecan Infinite M1000 PRO, TK Biotech, Warsaw, Poland) was used, and the IC_50_ value indicating the sample concentration at which 50% inhibition of ACE activity was determined by using linear regression analysis of logarithmic plots. At least 3 replicates for each standard solution were conducted. Captopril (0.1 μM solution in water), which is used therapeutically as an antihypertensive agent, served as the positive control.

### 4.4. Determination of the Acetylcholinesterase Inhibitory Activity of Flavone C-Monoglucosides Assay

The AChE inhibitory activity was evaluated following the methodology adapted from Eldeen et al. [85], and details of the procedure were provided recently by Zielińska et al. [86]. Galanthamine (0.5–50 μg/mL in water) served as the positive control, while water was used as the negative control. All measurements were conducted using a microplate reader (Tecan Infinite M1000 PRO, TK Biotech, Warsaw, Poland). The IC_50,_ indicating the concentration of the compound at which AChE activity is inhibited by 50%, was determined through linear regression analysis, with a coefficient of determination (R^2^) ranging from 0.805 to 0.999, based on triplicate measurements. At least 3 replicates for each standard solution were carried out.

### 4.5. Measurement of the Inhibitory Activity of Flavone C-Monoglucosides Towards Advanced Glycation End Products (AGEs) Formation

The inhibitory activity against AGEs formation was performed in the bovine serum albumin (BSA)/glucose and BSA/methylglyoxal (MGO) model systems as described recently by Zielińska et al. [86]. Standards of OR, hOR, VT, iVT, and Q were initially dissolved in a small volume of DMSO and then in phosphate buffer (0.1 M, pH 7.4) to obtain 1 mM (DMSO/phosphate buffer; 1:5; *v*/*v*). The positive control aminoguanidine (1 mM AG) was used [87]. The percentage (%) of inhibition was obtained from three repetitions (n = 3). The IC_50,_ indicating the concentration of the compound at which formation of AGEs is inhibited by 50%, was determined through linear regression analysis.

### 4.6. Statistical Analysis

Results are given as the average ± standard deviation (SD). The one-way analysis of variance (ANOVA) was used for the analysis of significant differences in the multifaceted biological activities of flavone *C*-monoglucosides (*p* < 0.05) (GraphPad Prism version 9 for Windows, GraphPad Software, San Diego, CA, USA). The correlation analysis was performed, and the Pearson correlation coefficient was calculated.

## 5. Conclusions

In this study, OR, hOR, iVT, and VT showed multifaceted biological activities. The OR and hOR showed high ACE and AChE inhibitory activity, whereas all compounds were strong inhibitors of AGEs formation. The rank of the ACE inhibitory activity in comparison to quercetin was Q > hOR > OR > VT > iVT, thus indicating the importance of the structure–antioxidant activity relationship. The order of the AChE enzyme inhibitory activity was hOR > OR > iVT > VT > Q, thus indicating for weak relationship with the chemical structure. The flavone *C*-monoglucosides, in comparison to quercetin, displayed strong inhibitory capacity on the AGEs formation as the rank of anti-AGEs activity in BSA/glucose and BSA/MGO model systems was hOR > OR > iVT > Q > VT > AG. The provided ranks clearly indicated the impact of the antioxidant potential and chemical structure on the multifaceted biological activities of flavone *C*-monoglucosides. The positive correlation was found between IC_50_ of OR, hOR, iVT, VT, and Q obtained in the BSA/glucose and BSA/MGO model system and their E_pa_ values provided by DPV (r = 0.546 and r = 0.580, respectively). No correlation was found between their ACE and AChE inhibition activities. The data on the inhibition of ACE and AChE activity and advanced glycation end-products (AGEs) formation are important against hypertension, Alzheimer-type dementia, and diabetic complications, respectively. The results expand our knowledge on the multifunctional activity of biologically active compounds of plant origin, which can be useful in dietotherapy and supporting the drug’s action. Orientin and homoorienin represent the most attractive flavone *C*-monoglucosides due to their high multifaceted activities.

## Figures and Tables

**Table 1:** The anodic peak potentials (E_pa_) and antioxidant activity of selected flavone *C*-monoglucosides in comparison to quercetin (Q) were provided by the differential pulse voltammetry technique.

Compound	$\mathrm{Anodic}\;\mathrm{Peak}\;\mathrm{Potential}\;E_{pa}$ (mV)			Antioxidant Activity (mM Trolox)
	$E_{pa1}$	$E_{pa2}$	$E_{pa3}$	
Orientin (OR)	345 ± 6 ^b^	1020 ± 9 ^a^	-	3.00 ± 0.14 ^b^
Homoorientin (hOR)	336 ± 5 ^b^	960 ± 9 ^b^	-	3.24 ± 0.15 ^b^
Vitexin (VT)	925 ± 8 ^a^	-	-	0.93 ± 0.07 ^c^
Isovitexin (iVT)	926 ± 8 ^a^	-	-	1.06 ± 0.07 ^c^
Quercetin (Q)	255 ± 7 ^c^	510 ± 8 ^c^	1060 ± 10	3.81 ± 0.19 ^a^

Data are expressed as means ± standard deviation (n = 3). Equation of the linear regression of Trolox concentrations (y = 133.29x + 8.21; R^2^ = 0.99) was used for the calculation of antioxidant activity. Means in a column followed by the different letters are significantly different (*p* ≤ 0.05) based on the one-way analysis of variance (ANOVA).

**Table 2:** The IC_50_ of selected flavone *C*-glucosides for ACE inhibition (µM).

Compound	Equation of the Linear Regression	IC_50_ (µM)
Orientin (OR)	y = 0.1807x + 27.908 R^2^ = 0.96	122.26 ± 2.66 ^c^
Homoorientin (hOR)	y = 0.2431x + 22.193 R^2^ = 0.98	114.39 ± 1.23 ^d^
Vitexin (VT)	y = 0.1701x + 26.982 R^2^ = 0.94	135.32 ± 2.45 ^b^
Isovitexin (iVT)	y = 0.1333x + 28.498 R^2^ = 0.96	161.31 ± 3.27 ^a^
Quercetin (Q)	y = 0.3831x + 27.285 R^2^ = 0.96	59.29 ± 0.48 ^e^
Captopril	y = 5008.1x + 20.632 R^2^ = 0.98	0.00586 ± 0.00004 ^g^
Glutathione (GSH)	y = 0.1337x + 44.429 R^2^ = 0.98	41.68 ± 3.45 ^f^

Data are expressed as means ± standard deviation (n = 3). Equation of the linear regression was used for IC_50_ calculation. Means in a column followed by the different letters are significantly different (*p* < 0.05) based on the one-way analysis of variance (ANOVA).

**Table 3:** The IC_50_ of *selected* flavone *C*-glucosides for AChE inhibition (µM).

Compound	Equation of the Linear Regression	IC_50_ (µM)
Orientin (OR)	y = 56.347x + 1.074 R^2^ = 0.99	0.868 ± 0.088 ^c^
Homoorientin (hOR)	y = 23.817x + 36.167 R^2^ = 0.81	0.581 ± 0.096 ^d^
Vitexin (VT)	y = 42.046x − 3.739 R^2^ = 0.99	1.278 ± 0.089 ^a^
Isovitexin (iVT)	y = 48.34x − 2.033 R^2^ = 0.99	1.076 ± 0.011 ^b^
Quercetin (Q)	y = 32.48x + 5.370 R^2^ = 0.99	1.374 ± 0.077 ^a^
Galanthamine	y = 834.84x + 14.195 R^2^ = 0.99	0.043 ± 0.008 ^e^

Data are expressed as means ± standard deviation (n = 3). The equation of the linear regression was used for IC_50_ calculation. Means in a column followed by the different letters are significantly different (*p* ≤ 0.05) based on the one-way analysis of variance (ANOVA).

**Table 4:** The IC_50_ inhibitory activity of selected flavone *C*-glucosides against AGEs formation (mM).

Compound	BSA-Glucose System		BSA-MGO System	
	Equation of the Linear Regression	IC_50_ (mM)	Equation of the Linear Regression	IC_50_ (mM)
Orientin (OR)	y = 0.0205x − 1.1218 R^2^ = 0.83	0.073 ± 0.005 ^c^	y = 0.0147x − 0.4095 R^2^ = 0.94	0.326 ± 0.019 ^e^
Homoorientin (hOR)	y = 0.017x − 0.8359 R^2^ = 0.70	0.014 ± 0.008 ^d^	y = 0.0149x − 0.3539 R^2^ = 0.986	0.391 ± 0.022 ^cd^
Vitexin (VT)	y = 0.0135x − 0.4199 R^2^ = 0.81	0.255 ± 0.016 ^b^	y = 0.0128x − 0.203 R^2^ = 0.98	0.437 ± 0.017 ^bc^
Isovitexin (iVT)	y = 0.0141x − 0.491 R^2^ = 0.82	0.214 ± 0.011 ^b^	y = 0.0139x − 0.3543 R^2^ = 0.94	0.341 ± 0.020 ^de^
Quercetin (Q)	y = 0.0164x − 0.5768 R^2^ = 0.86	0.243 ± 0.012 ^b^	y = 0.0141x − 0.2341 R^2^ = 0.99	0.466 ± 0.017 ^b^
Aminoquanidine (AG)	y = 0.0167x − 0.4031 R^2^ = 0.94	0.432 ± 0.036 ^a^	y = 0.0161x − 0.274 R^2^ = 0.99	0.531 ± 0.028 ^a^

Data are expressed as means ± standard deviation (n = 3). Equation of the linear regression was used for IC_50_ calculation. Means in a column followed by the different letters are significantly different (*p* ≤ 0.05) based on the one-way analysis of variance (ANOVA).

## Data Availability

The original contributions presented in this study are included in the article. Further inquiries can be directed to the corresponding author.
